# Multiscale patterns of isolation by ecology and fine-scale population structure in Texas bobcats

**DOI:** 10.7717/peerj.11498

**Published:** 2021-06-03

**Authors:** Imogene A. Cancellare, Elizabeth M. Kierepka, Jan Janecka, Byron Weckworth, Richard T. Kazmaier, Rocky Ward

**Affiliations:** 1 Department of Life, Earth, and Environmental Sciences, West Texas A&M University, Canyon, Texas, USA; 2 Department of Entomology and Wildlife Ecology, University of Delaware, Newark, DE, USA; 3 Department of Forestry and Environmental Resources, North Carolina Museum of Natural Sciences, Raleigh, North Carolina, USA; 4 Department of Biological Sciences, Duquesne University, Pittsburgh, Pennsylvania, USA; 5 Panthera, New York, New York, USA

**Keywords:** Bobcat, Gene flow, Landscape genetics, Redundancy analysis, Spatial autocorrelation, *Lynx rufus*, Isolation by ecology

## Abstract

Patterns of spatial genetic variation can be generated by a variety of ecological processes, including individual preferences based on habitat. These ecological processes act at multiple spatial and temporal scales, generating scale-dependent effects on gene flow. In this study, we focused on bobcats (*Lynx rufus*), a highly mobile, generalist felid that exhibits ecological and behavioral plasticity, high abundance, and broad connectivity across much of their range. However, bobcats also show genetic differentiation along habitat breaks, a pattern typically observed in cases of isolation-by-ecology (IBE). The IBE observed in bobcats is hypothesized to occur due to habitat-biased dispersal, but it is unknown if this occurs at other habitat breaks across their range or at what spatial scale IBE becomes most apparent. Thus, we used a multiscale approach to examine isolation by ecology (IBE) patterns in bobcats (*Lynx rufus*) at both fine and broad spatial scales in western Texas. We genotyped 102 individuals at nine microsatellite loci and used partial redundancy analysis (pRDA) to test if a suite of landscape variables influenced genetic variation in bobcats. Bobcats exhibited a latitudinal cline in population structure with a spatial signature of male-biased dispersal, and no clear barriers to gene flow. Our pRDA tests revealed high genetic similarity in similar habitats, and results differed by spatial scale. At the fine spatial scale, herbaceous rangeland was an important influence on gene flow whereas mixed rangeland and agriculture were significant at the broad spatial scale. Taken together, our results suggests that complex interactions between spatial-use behavior and landscape heterogeneity can create non-random gene flow in highly mobile species like bobcats. Furthermore, our results add to the growing body of data highlighting the importance of multiscale study designs when assessing spatial genetic structure.

## Introduction

Landscape heterogeneity has a powerful influence on genetic structure in nature, owing to the complex interaction between habitat preferences, resource distribution, movement capabilities, and underlying landscapes. Habitats are important for determining where organisms prefer to establish their home ranges and where they disperse; ultimately, shaping how landscape patterns influence gene flow. Much research has focused on how landscape heterogeneity influences genetic differentiation between populations or individuals, culminating in the field of landscape genetics (Manel et al., 2003; Storfer et al., 2010). Landscape genetics commonly focuses on how intervening habitat between areas influences connectivity, but patterns of spatial genetic variation can also be influenced by the environment at sample locations. Habitat preferences for certain environmental conditions can reduce gene flow regardless of intervening habitat, resulting in a pattern of isolation-by-ecology (IBE; Wang & Summers, 2010, Shafer & Wolf, 2013). Under an IBE scenario, genetic similarity is greater in more homogenous habitats than would be predicted under isolation by distance (IBD) (Sexton, Hangartner & Hoffmann, 2013). In IBD, genetic differentiation among individuals increases as geographical distances increase and mating tends to occur more frequently among neighbors (Wright, 1943). IBE patterns can occur with or without background patterns of IBD and result from a myriad of ecological processes (Sexton, Hangartner & Hoffmann, 2013), making studies on spatial genetic relationships useful for understanding how observed spatial genetic patterns are generated, and the role of specific environmental variables on influencing genetic variation, local adaptation, or dispersal limitations.

In the absence of IBD or obvious barriers, habitat preferences can result in cryptic genetic structure in even the most mobile, generalist species. Several studies have described the various effects of habitat heterogeneity on neutral genetic structure when space is not the dominant driving force of spatial genetic patterns (e.g., Edelaar et al., 2012; Sexton, Hangartner & Hoffmann, 2013; Wogan et al., 2019; Buskirk & Rensburg, 2020). For example, IBE patterns can result from biased dispersal, such as when a particular environment confers a fitness advantage, when dispersers avoid novel habitats, or when individuals exhibit natal-biased habitat dispersal (Wang & Bradburd, 2014). Despite the propensity for species with vagile mobility to exhibit either genetic panmixia or IBD at broad, range-wide scales, IBE patterns have been observed in widespread carnivores. Rueness et al. (2003) detected a geographically invisible barrier to Canada lynx gene flow that coincided with the ecological Continental and Atlantic regions of North America. Individual-specific habitat selection behavior (natal-biased habitat dispersal) facilitated cryptic population structure in coyotes (Sacks, Brown & Ernest, 2004; Sacks et al., 2005) and potentially maintained habitat-based genetic differentiation in bobcats (Reding et al., 2012). Predicting if IBE will occur is difficult in highly vagile species because both overall high connectivity and habitat-based gene flow can be detected in the same species (e.g., Reding et al., 2012; Kierepka & Latch, 2016a). Further, disparities between observed patterns in gene flow in populations of the same species can arise from examining landscape factors at differing spatial scales, as the landscape factors important for gene flow may not be fully encompassed or present at all spatial scales (e.g., Anderson et al., 2010; Short Bull et al., 2011; Hapeman et al., 2017; Kierepka et al., 2020).

Genetic variation in vagile species often is a function of spatial scale where habitat preferences and local adaptation influence gene flow at small spatial scales and high dispersal abilities impact broadscale population structure. Because the landscape variables impacting gene flow are not consistent across spatial scales (e.g., Anderson et al., 2010; Galpern, Manseau & Wilson, 2012; Hapeman et al., 2017), single-scale studies may overlook or underestimate important patterns of gene flow. As a result, recent studies have emphasized the importance of multiscale examinations of spatial genetic structure (Aylward, Murdoch & Kilpatrick, 2020; Burgess & Garrick, 2020; Kierepka et al., 2020). However, multiscale designs are less straightforward for populations of widespread, vagile, or generalist species due to their broad distribution, high abundance, and population connectivity (Reding et al., 2012; Kierepka & Latch, 2016b). Instead, scale in such species can be defined via behavioral data, specifically at a home range and dispersal scale. Home ranges may reflect fine-scale selection of habitat types and avoidance of barriers/unsuitable areas, and are often correlated with gene flow in single-scale studies (e.g., Riley et al., 2006; Musiani et al., 2007; Schwartz et al., 2009; Ruell et al., 2012). Regardless of these fine-scale patterns, long-distance dispersal occurs at much larger spatial scales for many widespread species, so habitat selection and fine-scale gene flow are unlikely to translate to broader spatial scales. Many species are also less selective in dispersal habitats (e.g., Kierepka & Latch, 2016a), which could have strong management implications for maintaining broadscale connectivity in increasingly human-dominated landscapes.

This study focused on multiscale landscape effects on gene flow in the bobcat (*Lynx rufus*), a vagile, generalist felid found throughout North America (Anderson, 1987). Despite the potential for high gene flow, examinations of landscape effects on bobcat genetic variation have revealed significant influences on genetic substructure across spatial scales, including sensitivity to roads at home range-sized spatial scales (Riley et al., 2006; Serieys et al., 2015), fragmentation and increased urban land use as barriers (e.g., Janečka et al., 2016; Kozakiewicz et al., 2019; Smith et al., 2020), and behavioral patterns of dispersal and philopatry in contiguous habitat (Janečka et al., 2006; Croteau, Heist & Nielsen, 2010). Importantly, bobcats are hypothesized to practice natal habitat-biased dispersal at forest-scrubland breaks within the midwestern United States, which resulted in habitat-based genetic differentiation (Reding et al., 2012). At a broader spatial scale, studies of bobcats have identified landscape characteristics and habitat composition (e.g., level of agriculture land use, percentage of forested cover) as constraints to gene flow at regional or continental scales (Croteau et al., 2012; Reding et al., 2013). Taken together, these findings suggest that relevant ecological relationships may not be consistent across spatial scales for any given population (Keeley et al., 2017); thus, a multiscale framework may facilitate a better interpretation of genetic variation in bobcats.

To examine multiscale patterns of gene flow in bobcats, we used ordination techniques to examine patterns of spatial genetic structure for Texas bobcats. Specifically, these analyses tested if bobcats exhibit a simple pattern of gene flow (i.e., IBD or panmixia) or a more complex association between genetic differentiation and site-specific environmental differences (IBE). Because the scale of landscape sampling can introduce varying features that may affect gene flow, we conducted multiscale analyses to test if IBE patterns differed based on spatial scale. Overall, bobcats’ high movement capability and generalist ecology could result in little genetic structure as expected in a relatively contiguous landscape. However, phylogenetic lineages of bobcats meet in the southern Great Plains along habitat gradients, a similar scenario to where bobcats exhibit habitat-based differentiation in the Midwestern United States (Reding et al., 2012). In the absence of obvious barriers, we predicted bobcats would exhibit high levels of gene flow, with fine-scale population structure reflecting sex-biased dispersal, a well-documented phenomenon in felids (Croteau, Heist & Nielsen, 2010; Janečka et al., 2006). We also expected bobcats to exhibit IBE patterns, with site-specific differences in landscape heterogeneity that differed by spatial scale, likely as a result of disparate habitat preferences and habitat availability at each scale. Ultimately, this study aims to identify multiscale relationships between landscape patterns and gene flow in bobcats, which can provide wildlife managers critical information on where to mitigate the population effects of landscape change (e.g., Thatte et al., 2018, Kaszta et al., 2019).

## Study Area

Our study area comprised five level III ecoregions across western Texas: High Plains, Rolling Plains, Edward’s Plateau, Trans-Pecos, and South Texas Plains (Omernik, 1987). Briefly, these ecoregions differ by habitat type, elevation, plant community structure, and topographic relief. The transitions in elevation, vegetation composition, and habitat types vary significantly both within and between these regions; for a detailed description see Blair (1950) and Omernik (1987). The landscape heterogeneity across western Texas has changed dramatically in the last two hundred years, including considerable expansion of urban and agricultural land use. Bobcats occur in all ecological zones of Texas (Schmidly, 2004) and the statewide population is estimated to be between 287,444–1,357,928 individuals (Roberts & Crimmins, 2010), but recent genetic evidence suggests that bobcats in southern Texas are negatively impacted by severe habitat fragmentation (Janečka et al., 2016).

### Sample collection and laboratory techniques

Tissue samples from 102 bobcats were collected between January 2013 and March 2015 primarily through opportunistic sampling of road-killed (*n = 2*) and fur-harvested animals (*n = 98*). Two additional samples were obtained from live animals, which were live-trapped with approval obtained from the West Texas A&M University Institutional Animal Care and Use Committee (IACUC # 04-12-12). Individuals were sexed (30 males, 61 females, 11 unknown) and classified as adult (*n = 93*), juvenile (*n = 2*), or unknown age (*n = 7*). DNA extractions on all samples were conducted using a modified Gentra Puregene tissue kit protocol (QIAGEN Corporation, Valencia, CA, USA). The primary modification concerned tailoring the amount of elution buffer based upon the quantity of DNA after the final EtOH wash, as revealed by visualization in a 1% agarose gel following electrophoresis. We genotyped all samples at 9 microsatellite loci shown to be variable in other bobcat genetic studies (FCA 26, FCA 43, FCA 45, FCA 77, FCA 82, FCA 90, FCA 96, FCA 132; Menotti-Raymond et al., 1999, Lc 120; Carmichael, Clark & Strobeck, 2000).

Polymerase chain reactions (PCR) were conducted in 12.5 ul volume reactions containing GeneMate Taq 2X Mastermix (GeneMate) with 2.5 mM MgCl^2^, Well-Red fluorescently-labeled oligonucleotide (Sigma-Genosys, The Woodlands, TX, USA), 10 uM forward primer, 10 uM reverse primer, and 20–325 ng DNA template. PCR reaction thermal cycling conditions for all 9 microsatellites included an initial denaturing step of 94 °C for 1 min, 10 cycles of 94 °C for 15 s, 53 °C for 15 s and 72 °C for 45 s, followed by 50 cycles of 89 °C for 15 s, 53 °C for 15 s and 72 °C for 45 s and a final extension of 72 °C for 30 min (Janečka et al., 2006). The denaturing temperature was lowered to 89 °C after 10 cycles to decrease the amount of Taq inactivated by the high temperature of each denaturing step (Menotti-Raymond et al., 1999). All amplifications were performed on an Eppendorf Mastercycler (Eppendorf, Hamburg, Germany). Samples were fractionated with a Beckman Coulter CEQ8000 DNA Analyzer (Beckman Coulter, Indianapolis, IN, USA), genotyped using the CEQ8000 software, and allele calls confirmed by visual inspection of electropherograms.

### Genetic diversity and population structure

We quantified genetic metrics among 102 bobcats using number of alleles (*A*), observed and expected heterozygosity (*H*_O_ and *H*_E_, respectively), *F*_ST_ and *F*_IS_ estimates (population subdivision and inbreeding coefficients, respectively) with GenAlEx 6.5.03 (Peakall & Smouse, 2006, 2012). Deviations from Hardy-Weinberg equilibrium (HWE) and linkage disequilibrium were calculated in genepop (Raymond & Rousset, 1995; Rousset, 2008) using a corrected alpha for multiple tests (*α* = 0.0011; Rice, 1989).

We characterized population genetic structure for all bobcats in western Texas using the non-spatial, Bayesian clustering approach in structure (Pritchard, Stephens & Donnelly, 2000), which uses individual genotypes to determine the optimal number of populations (*K*) and quantifies levels of admixture between putative clusters (Pritchard, Stephens & Donnelly, 2000; Rosenberg et al., 2002). We performed 10 independent runs of *K* = 1 to 10 with 1,000,000 Markov Chain Monte Carlo (MCMC) steps and 300,000 burn-in steps under the admixture, correlated allele frequency model. The optimal *K* among the tested values was determined by visual examination of the likelihood scores in structure harvester (Earl & vonHoldt, 2012) using the Δ*K* statistic (Evanno, Regnaut & Goudet, 2005) because likelihood values plateau and variances among runs grow larger when values of *K* are above the optimum (Pritchard, Stephens & Donnelly, 2000). Individuals were assigned to each putative cluster based on their highest ancestry coefficient (*q*), a value that represents the proportion of an individual’s genome that belongs to each cluster structure bar plots were created in structure plot (Ramasamy et al., 2014).

To complement our structure analysis, we also conducted exploratory multivariate analyses, including discriminant analysis of principal components (DAPC), using *adegenet*, version 2.1.1 (Jombart, 2008) in R, version 1.1.463 (2019). We used the find.clusters() function to determine the number of clusters (*K*) de novo, retaining all principal components (PCs) to infer a range of possible clusters. We selected the optimal *K* as that with the lowest BIC value. The optimal number of PCs to use in the DAPC was determined using the xval method.

In addition to identifying the number of genetic clusters across our study area, we also tested for the presence of IBD via a simple Mantel test. Simple Mantel tests quantify the correlation between matrices of pairwise genetic and Euclidean distances. We used GenAlEx to calculate Euclidean and pairwise genetic distance (default genetic distance in GenAlEx). Statistical significance was assessed with 10,000 permutations.

### Spatial patterns and sex-biased dispersal

In addition to tests for IBD, we used spatial autocorrelations to detect departures from random mating at pre-defined distance intervals. For species that exhibit sex-biased dispersal, spatial autocorrelation is predicted to be weaker for the sex that exhibits greater dispersal frequency (i.e., philopatry). Like many mammals (e.g., Tucker et al., 2017; Oliveira et al., 2018), bobcats exhibit male-biased dispersal, with females being philopatric (Janečka et al., 2006, 2007; Croteau, Heist & Nielsen, 2010). We used spatial autocorrelation analysis as implemented in genalex to test for spatial genetic structure and sex-specific differences in bobcats. We predicted that females in close proximity would exhibit positive spatial autocorrelation (i.e., be more genetically related than expected by chance) due to philopatry, while males would exhibit random spatial autocorrelation consistent with dispersing from their natal range. GenAlEx computes an autocorrelation coefficient (*r*) using matrices of pairwise geographic and squared genetic distances between genotypes across all microsatellite loci to detect departures from random mating (i.e., *r* = 0), then performs both a permutation and bootstrap test to assess significance of *r*-values within pre-defined distance intervals (Peakall & Smouse, 2006, 2012). Philopatric individuals separated by small geographic distances are expected to be more genetically similar, thus, we used spatial autocorrelations to detect departures from random mating within 5 km distance categories (Kierepka & Latch, 2016a). Significance in all spatial autocorrelation analyses were calculated based on 10,000 permutations and bootstraps.

### Landscape variables

Defining relevant spatial scales in highly mobile species often relies on either expert opinion or is an artifact of opportunistic sampling, so we calculated landscape variables at two biologically relevant spatial scales: a fine spatial scale based on bobcat home range sizes, and a broad spatial scale based on reported dispersal distances. For the fine spatial scale, we calculated landscape variables within a 2.69 km radius buffer around each point. The distance was based on the mean of published home range sizes in the southern Great Plains (Rolley, 1985, Kamler & Gipson, 2000, Elizalde-Arellano et al., 2012). For the broad spatial scale, we buffered sample locations with a radius of 5.58 km; the resulting buffer polygons represented the mean of the typical dispersal distances for bobcats reported from the literature (Chapman & Feldhammer, 1982). These buffer polygons were intersected with GIS layers for each environmental variable to determine scale-specific information for each sample.

In total, both spatial scales were examined using nine environmental variable classes (Land Use Land Cover Categories, Stream Density, Road Density, Railroad Density, Annual Precipitation, Maximum Temperature, Minimum Temperature, Ecoregion, and Vegetation Composition). Geospatial data for each environmental variable class was pulled from the Texas Natural Resources Information System (TNRIS). We selected variables from literature that found correlations between bobcat behavior and ecology and a particular landscape variable, or were previously shown to influence gene flow in other meso-carnivore species (e.g., Reding et al., 2012, Kierepka & Latch, 2016a). Each environmental variable category was represented by a GIS layer, which was used to calculate proportions and densities of variables within individual buffer polygons. Thus, each individual bobcat had a point estimate of our suite of landscape variables at both a fine (home range-sized polygon) and broad (dispersal-sized polygon) scale.

At both spatial scales, we used Patch Analyst 3.1 (Rempel, Kaukinen & Carr, 2012) to calculate fragmentation statistics (number of patches, mean patch size, mean patch edge, patch density, edge density, total edge, and Shannon’s diversity index of patch size) for the Land Use Land Cover Categories (Table S1). All spatial analyses were performed using ArcView 3.3 (Environmental Systems Research Institute, Redlands, CA, USA). To avoid correlation among explanatory variables, we used Pearson’s correlation analysis with the corr.test function in the base package of R for all landscape variables at both spatial scales, and removed variables with *r* > 0.7. For both spatial scales, we also removed variables with 50% 0 s or more (i.e., variables with low proportions in the buffer polygons across all cats).

### Landscape effects on genetic variation

Ordination techniques are especially useful in landscape studies due to their ability to identify the combination of landscape variables that influence genetic variation, and can overcome assumptions of linearity that characterize distance-based methods (Kierepka & Latch, 2015). We used redundancy analysis (RDA; Legendre & Legendre, 1998) models to disentangle the effect of landscape variables and geographic distance on bobcat genetic variation at two spatial scales. RDA is a multivariate technique analogous to linear regression, and is a powerful test for analyzing spatial genetic structure because it reduces type I errors when comparing landscape genetic relationships and can perform multi-model comparisons (Legendre & Fortin, 2010; Kierepka & Latch, 2015). Partial redundancy analysis (pRDA) first removes the effect of one or more explanatory variables on a set of response variables (in this case, controlling for spatial location), followed by a standard RDA. This method is useful for examining how landscape factors impact gene flow independent of the spatial structure that species and environmental variables may share (Borcard, Legendre & Drapeau, 1992).

We used pRDA to examine the relationship between our landscape variables and an individual-based measure of genetic variation at both spatial scales. To apply pRDA, we initially performed a spatial principal component analysis (sPCA; Jombart et al., 2008) using bobcat microsatellite genotypes and retrieved the linearized, spatially lagged scores of the first two principal components as response variables in the pRDA. sPCA provides principal components scores that explain non-spatial patterns of genetic variation and the spatial autocorrelation structure among individual genotypes (Jombart et al., 2008). We performed sPCA in the R package *adegenet* (Jombart, 2008). The connection network used to calculate sPCA scores requires a cutoff if a dispersal limitation is present. Given their continuous distribution throughout Texas, and because theoretically a bobcat could move across our study site, we used a distance-based connection network to ensure all cats were connected. Axes with the highest eigenvalues are considered the most important because they explain the most variation. Scores from axes that had the highest eigenvalues were separated in a screeplot and plotted in ArcMap to examine geographic structuring within the sPCA.

We performed all RDA analyses in the R package *vegan* (Oksanen et al., 2008) at both scales using the proportions of landscape and environmental variables calculated for polygons at each buffer size. Our pRDA analysis included three steps. An initial marginal test included all predictor variables, followed by stepwise model selection to identify the variables that best explained genetic differences among individuals (the function ‘ordistep’). After significance testing using ‘anova.cca’, each marginal model containing significant landscape variables was then rerun without geographic coordinates to evaluate how much variation was explained by the landscape and environmental factors alone.

## Results

### Genetic diversity and population structure

We observed a heterozygote deficiency over all loci in a global analysis (*F*_IS_ = 0.115), which was high compared to another study that used the same loci (*FCA 43*, *FCA 45*, *FCA 77*, *FCA 90*; Croteau, Heist & Nielsen, 2010). Deviations from Hardy–Weinberg equilibrium are expected if there is structure within the data set. All loci were highly polymorphic with 8-16 alleles per locus (mean = 11.78, Table 1), and no evidence of linkage disequilibrium was found in any locus-pairs. Tissue samples yield high-quality DNA with unambiguous allele peaks and we did not consider allelic dropout to influence the observed deviations from Hardy–Weinberg. Because none of our analyses outside of structure assume Hardy–Weinberg, we retained all loci in subsequent analyses to maximize explanatory power.

**Table 1:** Locus-specific summary of genetic variation for *n* = 102 bobcats across western Texas.

*Locus*	A	*H*_E_	*H*_O_	*F*_IS_
FCA 26	12	0.833	0.737	0.115
FCA 43	10	0.794	0.747	0.058
FCA 45	11	0.799	0.670	0.162
FCA 77	13	0.843	0.798	0.054
FCA 82	15	0.873	0.849	0.027
FCA 90	8	0.818	0.711	0.131
FCA 96	10	0.835	0.726	0.131
FCA 132	11	0.57	0.789	0.079
Lc 120	16	0.856	0.613	0.284
Overall	11.78	0.836	0.753	0.115

**Note:**

Abbreviation: Metrics included are the number of alleles per locus (A), observed heterozygosity (*H*_O_), expected heterozygosity (*H*_E_), and inbreeding coefficients (*F*_IS_) for each locus.

**Figure 1:**
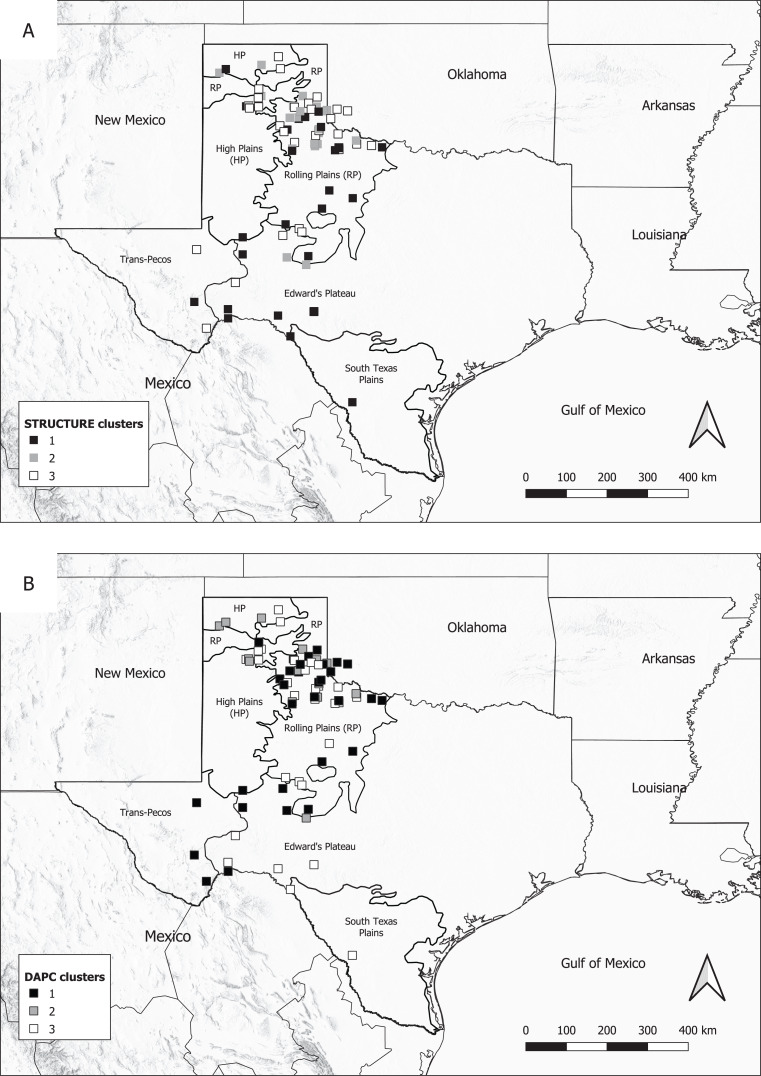
Distribution of genetic clusters inferred from *q*-values in Structure (A) and DAPC analysis (B). For both analyses, all 102 individuals were assigned to one of three genetic clusters based on the 9 microsatellite loci. However, when the highest *q*-values for each sample were mapped (A), the three putative clusters from structure did not exhibit a spatial pattern (e.g., clear partitioning), suggesting *K* = 3 is not biologically meaningful. Upon visual inspection, the overlap of the discriminant functions from DAPC (B), combined with the random spatial arrangement of the STRUCTURE *q*-values, suggests instead two genetic groups exhibiting a weak latitudinal cline.

In the structure analysis, *K* = 3 was the optimal number of populations (Δ*K* = 18.32, average likelihood: 3,482.51), and the spatial arrangement of the highest *q*-values suggested a weak latitudinal cline of the three clusters (Fig. 1; see Fig. S2 for structure *q*-plots at *K* = 2–5 and Fig. S3 for structure Harvester outputs). Pairwise *F*_ST_ values between the three putative clusters from structure were low, but significant (*F*_ST_ = 0.009, 0.011, 0.032, *P* = 0.010–0.001). Visual examination of individual assignments to each cluster showed a random pattern, with no correlation to geographic location. Latch et al. (2006) found that structure may provide false certainty regarding *K* when *F*_ST_ is low (*F*_ST_ < 0.05). Taken together, *K* = 3 may not reflect true population structure. For DAPC analysis, the optimal number of clusters was found to be 3, and the optimal number of PCs retained for analysis was 20 (Fig. S4 for BIC plot). DAPC analysis suggested that bobcats in western Texas form three groups with low levels of genetic differentiation, but the discriminant functions (Fig. S5) showed considerable overlap for groups 1 and 2 suggesting bobcats form two groups instead of three (Fig. 1).

### Spatial patterns and sex-biased dispersal

**Figure 2:**
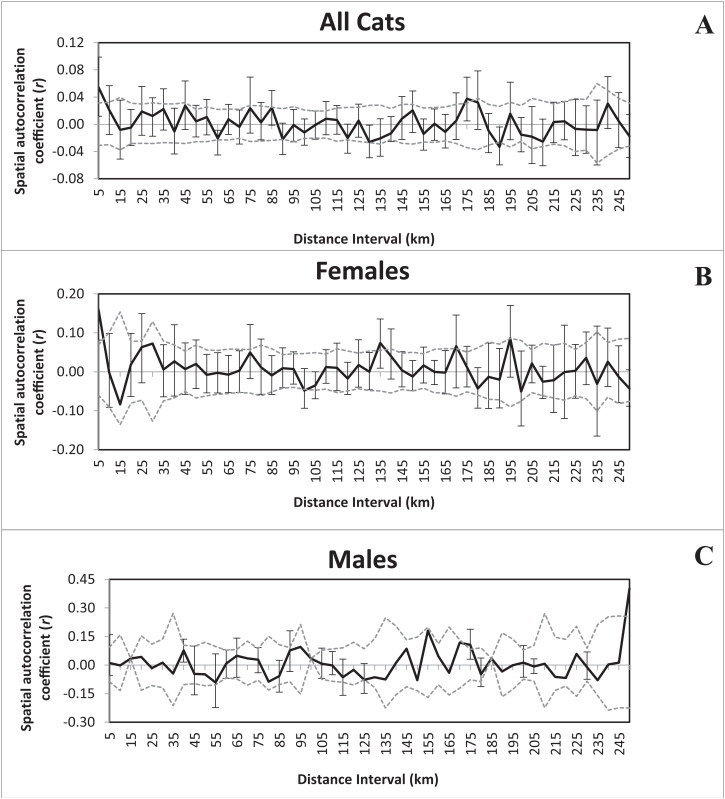
Correlograms illustrating the influence of distance on spatial autocorrelation for all 102 bobcats (A), females (B), and males (C). Gray upper and lower errors bars bound the 95% confidence interval about r by bootstrap resampling (shown in black). Distance intervals were set to an even distance of 5 km. Genetic distances between proximate individuals (<5 km) were smaller than expected under panmixia in spatial autocorrelation for all cats. When analyzed by sex, females exhibited strong correlations between genetic and geographic distance (< 5 km, r = 0.160, *P* = 0.001), whereas males were random, indicating support for philopatry in female bobcats.

The Mantel test did not show a significant correlation between matrices of genetic and geographic distances (*r* = 0.031, *P* = 0.250). However, we observed support for fine-scale spatial autocorrelations among all bobcats. For all 102 cats, autocorrelation coefficients between proximate individuals were significantly larger than by chance (<5 km, *r* = 0.055, *P* = 0.002; Fig. 2). When we analyzed spatial autocorrelation by sex, females exhibited strong correlations between genetic and geographic distance (<5 km, *r* = 0.160, *P* = 0.001), whereas males were random (Fig. 2).

### Landscape effects on genetic variation

**Figure 3:**
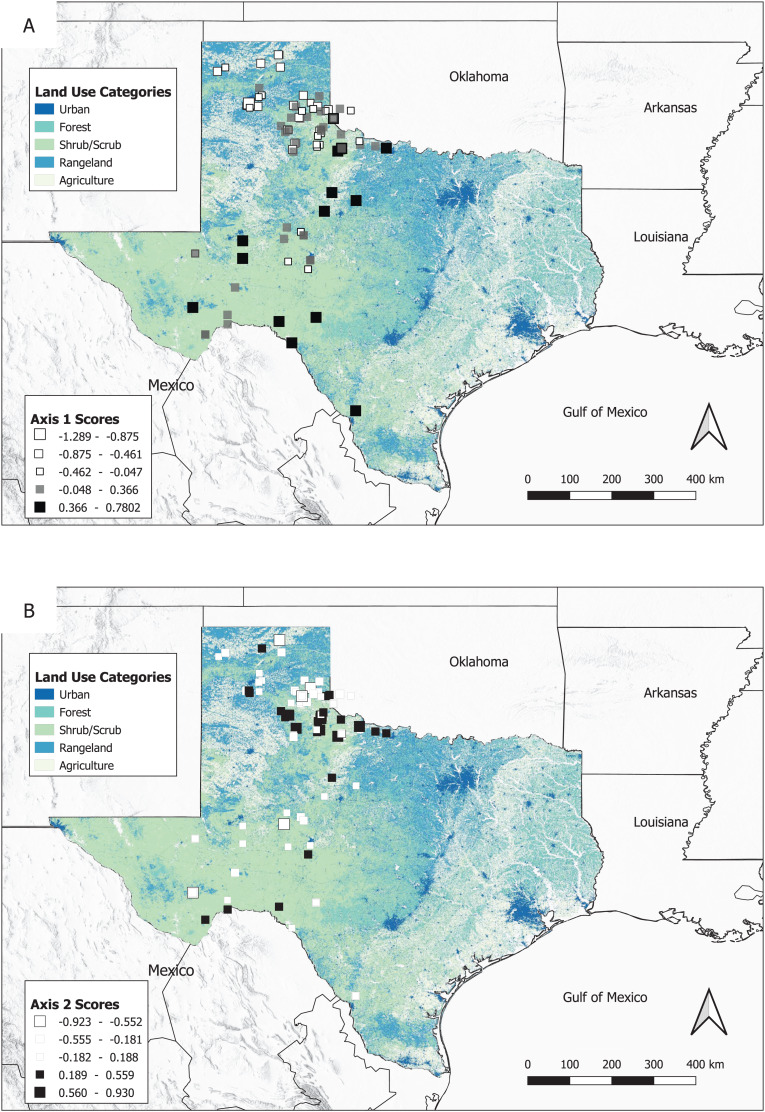
Map of land use categories with geographic locations of bobcat samples featuring spatially lagged scores for the first two sPCA axes. At the fine scale, scores from Axis 1 were correlated with the proportion of Herbaceous Rangeland, and at the broad scale, Mixed Rangeland and Agriculture (A). No environmental variables were significantly correlated with scores from Axis 2 (B). White represent positive sPCA scores (black and dark gray represent negative values). More extreme values in sPCA axes are displayed with larger squares.

Spatial PCA axes (Fig. 3) revealed a latitudinal cline across western Texas (Axis 1), with some genetic similarity in the Rolling Plains ecoregion revealed by Axis 2, though this pattern may be an artifact of uneven sampling. Both axes had signatures of spatial autocorrelation (Moran’s *I* = 0.493 and 0.470; Fig. S1). The correlations between sPCA scores and habitat types across western Texas appears to be scale dependent. For each habitat type retained as significant in the pRDA, greater amounts of each habitat type were associated with higher PC scores on Axis 1, which could indicate that these habitat types increase bobcat genetic differentiation (i.e., those in agriculture are more genetically different than those in less agriculture). At the fine scale, the land use category of herbaceous rangeland (*F*_1, 101_ = 11.7379, *P* < 0.001) was retained after model selection, and was associated with spatially lagged scores for sPCA Axis 1, suggesting individuals living in herbaceous rangeland are more genetically similar. The land use categories labeled urban, agriculture, mixed rangeland, and stream density were insignificant variables and not retained at the fine scale. The adjusted R-squared value, which represents the proportion of variation explained after removing variability due to the conditional terms (here, latitude and longitude to control for IBD), was low (0.096).

In contrast, at the broad spatial scale, the land use categories of mixed rangeland (*F*_1, 101_ = 24.9527, *P* < 0.001) and agriculture (*F*_1, 101_ = 8.2703, *P* < 0.01) were significant and retained as variables after model selection. Mixed rangeland was associated with spatially lagged sPCA scores for Axis 1, and agriculture was also detected as a potential influence on spatially lagged sPCA scores for Axis 1. No variables were significantly associated with Axis 2. The variables included in the broad spatial scale pRDA were the same as in the fine spatial scale pRDA with the addition of the vegetation type mesquite juniper bush. The adjusted R-squared value at the broad scale explained a higher proportion of the genetic variation than the fine scale (0.236).

## Discussion

Overall, our study found non-random gene flow in bobcats at both spatial scales despite bobcats being a highly mobile generalist. These life history traits likely drove the weak genetic structure across the entire study area as evidenced by ambiguous clustering results from structure and DAPC. Although our genetic dataset revealed low levels of genetic differentiation across the entire study area, we recorded scale-dependent evidence of spatial genetic structure. Geographic distance was important at a fine scale due to sex-biased dispersal, but no IBD was found across the study area. Furthermore, ordination analyses showed differing landscape variables explained the significant associations between landscape heterogeneity and bobcat genetic variation across fine and broad spatial scales. Thus, our analyses suggest weak IBE because landscape variables were significantly associated with similar sPCA scores in bobcats.

### Population structure and sex-biased dispersal

Bobcats exhibited clinal population genetic structure. Spatial examination of the *q*-values in both structure and a plot of the discriminant functions from DAPC identified two groups that were roughly partitioned into a north group and south group. This genetic subdivision corroborated the latitudinal cline apparent in the sPCA. Both axes in the sPCA had relatively strong signatures of spatial autocorrelation, particularly in Axis 1, demonstrating that geographic distance influenced bobcat gene flow at small spatial scales. Specifically, the scores from the first principal component revealed a latitudinal (north-south) cline, differentiating bobcats in the northern portion of western Texas from cats in the southern half. This cline was similar to that detected by Reding et al. (2012), where bobcats in the northern portion of western Texas were more genetically similar to one another than to cats in the southern portion of the state. The latitudinal gradient we observed in the spatially lagged scores from the sPCA may reflect stepping-stone dispersal movements (Kimura & Weiss, 1964) by bobcats, but the spatial extent at which we sampled may not have been adequate to detect a decrease of genetic correlation with distance (i.e., IBD).

As predicted, we found support for fine-scale genetic structure consistent with sex-biased dispersal in bobcats (Croteau, Heist & Nielsen, 2010), which may explain why all but one locus was out of Hardy–Weinberg equilibrium. Similar patterns have been observed in coyotes (Sacks, Brown & Ernest, 2004) and European grey wolves (Pilot et al., 2006), both of which were hypothesized to exhibit natal-habitat biased dispersal. Deviations from HWE could be further driven by philopatry in female bobcats because field observations (Janečka et al., 2006; Rodgers et al., 2015; Beugin et al., 2016) and our spatial autocorrelations strongly support philopatry in female bobcats. Thus, females may be driving the genetic structure and deviations from HWE found in our analyses, but additional investigations are needed to differentiate between simple philopatry or natal-biased habitat dispersal.

### Landscape effects on genetic variation of bobcats in western Texas

Our ordination analysis found that patterns of landscape effects on genetic variation differed by spatial scale, which highlights the importance of multiscale designs to gain a more holistic view of landscape effects on genetic variation. At the fine scale, our analysis detected herbaceous rangeland as a potential influence on patterns in the sPCA Axis 1. Herbaceous rangeland is dominated by naturally occurring and modified areas that include tall and short grasses and forbs as their principal cover (Anderson et al., 1976). The association between genetic variation and herbaceous rangeland suggests that bobcats within this habitat are more genetically similar than those found outside. Rangeland habitats generally comprise more diverse plant communities than agriculture, and may offer more resources for bobcats. For example, Taylor (2017) found that bobcat populations in contiguous rangeland habitats in south Texas showed panmixia, with bobcats preferring to disperse into rangeland habitat. As a result, herbaceous rangeland may be a preferred habitat for bobcats in the region sampled and increase functional connectivity in western Texas.

Mixed rangeland and agriculture were dominant influences on genetic variation at the broad spatial scale. This contrast with the fine scale emphasizes how spatial scale can impact the relationship between gene flow and landscape structure (Cushman & Landguth, 2010). Mixed rangeland contains a higher proportion of herbaceous and shrub or brush rangeland species than herbaceous rangeland, resulting in more cover which may be suitable for bobcats (Anderson et al., 1976; Reding et al., 2013). Because correlations between genetic variation and rangeland types were observed at both spatial scales, it appears to reinforce the observation that bobcats seek out habitats with high degrees of heterogeneous composition (Reding et al., 2013; Clare, Anderson & Macfarland, 2015). While rangeland activities that enhance livestock production often coincide with predator removal and have negative impacts on several mesocarnivore populations (Krausman et al., 2011), in Texas, where 97% of the land is privately owned, land management practices that promote diverse plant communities and heterogeneous habitat types may increase structural and functional connectivity of bobcats.

Agriculture had a weak influence on genetic variation at the broad scale. Bobcats tend to avoid large homogenous agricultural patches (Nielsen et al., 2018), and one study on bobcat response to spatial heterogeneity found bobcats avoided agriculture, but it was not a barrier to gene flow (Reding et al., 2013). Similarly, Hilty & Merenlender (2004) found that bobcats use riparian corridors to move through agricultural land. It is possible that agricultural habitat is correlated with genetic variation in bobcats via bobcats dispersing through sub-optimal habitats or through proximity to higher quality habitat such as rangeland. Alternatively, faster movements through agriculture during dispersal could result in reduced differentiation among individuals within agricultural and surrounding habitats (Kierepka et al., 2020). The genetic similarity among cats in the Rolling Plains ecoregion revealed by the spatially lagged scores in Axis 2 may reflect this scenario, as this ecoregion is adjacent to extensive agricultural land use (Fig. 3B). Corroborating movement data from telemetry studies with genetic data could help determine the exact influence of agriculture on bobcat gene flow.

Overall, landscape effects on bobcat genetic variation were relatively weak in our study area, which argues against strong habitat-biased dispersal and IBE in our study area. Certainly, other environmental factors could influence genetic variation like prey preference and abundance, which has also been shown to influence genetic structure (Pilot et al., 2006; Carmichael et al., 2007) and habitat-specific genetic subdivisions (Carmichael et al., 2007). For bobcats in this study, the associated habitat and vegetation transitions may have been too gradual across the ecoregions of western Texas (Fig. 1) for strong IBE or were overshadowed by the strong philopatry of female bobcats. Bobcats are known to be sensitive to extreme habitat fragmentation and broad habitat breaks (e.g., Riley et al., 2006; Reding et al., 2012; Poessel et al., 2014), so our study area may not reflect these extreme landscape changes. More intensive sampling and broader sampling distribution is needed to determine whether functional responses are reflected in genetic data for Texas bobcats. Molecular markers like SNPs would be useful for examining habitat-specific divergences of bobcats in this area, particularly given that Reding et al. (2012) identified the southern Great Plains as a zone of secondary contact for bobcat lineages.

Bobcats exhibit considerable behavioral plasticity, making it difficult to predict if landscape heterogeneity predicts genetic variation (i.e., IBE) and what scale is most relevant to examine such effects. Our study underscores how fine-scale analyses across an environmental gradient at multiple scales can identify unique landscape factors important for spatial genetic structure. In particular, site-specific habitat differences based on habitat are more likely to reveal important environmental variables that generate habitat-based genetic breaks as predicted by habitat-biased dispersal or local adaptation. Despite being a highly vagile species, the ecological and social determinants of bobcat gene flow in Texas appear to be scale-dependent, and the processes resulting in the IBE patterns are complex. These results likely reflect linkages among different scales of landscape composition and configuration, which is relevant to bobcat management in Texas. When combined with multiscale analyses, IBE methods can address the challenges of using landscape and genetic data to identify meaningful ecological patterns for management (Balkenhol et al., 2009).

## Supplemental Information

10.7717/peerj.11498/supp-1 Supplemental Information 1 Spatial variance of the eigenvalues from the spatially lagged PCA. (A) Eigenvalues of sPCA (denoted λi with i = 1, …, r, where λ1 is the highest positive eigenvalue, and λ93 is the highest negative eigenvalue) according their variance and Moran’s I A B components. (B) Positive eigenvalues (on the left) correspond to global structures, while negative eigenvalues (on the right) indicate local patterns.

10.7717/peerj.11498/supp-2 Supplemental Information 2 STRUCTURE outputs for K = 2-5 for bobcats in western Texas. Structure of bobcat clusters (K) in western Texas revealed by Bayesian analysis implemented in STRUCTURE. Each individual is represented by a vertical bar broken into different colored genetic clusters and arranged by latitude, with length proportional to the assignment probability to each cluster. Analysis of 102 individuals, with possible numbers of clusters ranging from 2–10, indicated that the most likely number of clusters was 3. Bar plots created in STRUCTURE PLOT (Ramasamay et al. 2014).

10.7717/peerj.11498/supp-3 Supplemental Information 3 Statistical output from Structure Harvester for bobcat clusters. Statistical output from Structure Harvester suggesting [A] optimal K with ΔK and [B] LnP(D).

10.7717/peerj.11498/supp-4 Supplemental Information 4 BIC plot from DAPC analysis. Bayesian information criterion (BIC) plot for DAPC between 1 and 10 showing an elbow with an arrow at K = 3.

10.7717/peerj.11498/supp-5 Supplemental Information 5 DAPC scatterplot and discriminant functions revealing cluster assignments for 102 bobcats. [A] Discriminant Analysis of Principal Components (DAPC) scatterplot drawn using nine microsatellites across 102 individual bobcats in the R package *adegenet*. Dots represent individuals, with colors denoting cluster assignment. [B] A plot of the individual densities against the first discriminant function retained show that the greatest proportion of variation lies with it. Colors correspond to cluster assignments, with significant overlap between clusters 1 and 3, suggesting two groups instead of 3.

10.7717/peerj.11498/supp-6 Supplemental Information 6 Land use and environmental predictor variables retained for RDA at two spatial scales. We intersected GIS layers for each variable with a buffer polygon at both spatial scales and calculated the proportion of each variable within each buffer polygon. For the linear variables (Stream Density, Railroad Density, and Road Density), we divided total variable lengths by the total hectares of the buffer polygons and converted to km/km2. For stream density, we included streams above an order of magnitude of 5 given the likelihood that anything below this cutoff would not be a significant barrier to bobcat movement. Maximum and minimum temperature reflected the weighted average of maximum minimum temperatures within each polygon. We calculated annual precipitation to a weighted average by intersecting this layer with buffer polygons and adding the products of the number of hectares at each rain average value (in) and divided by the total hectares of each buffer polygon. To determine which variables to use in the partial redundancy analysis (RDA), variables were selected from among nine environmental classes following Pearson’s correlation, and selected if *r* > 0.7.

10.7717/peerj.11498/supp-7 Supplemental Information 7 R code for spatial analyses. R code for sPCA, DAPC, RDA analyses of raw data.

10.7717/peerj.11498/supp-8 Supplemental Information 8 Genetic data for 102 bobcats. Raw data for genetic analyses, including genotypes, geographic data, and output for descriptive statistics using GenAlEx.

10.7717/peerj.11498/supp-9 Supplemental Information 9 Genepop file for 102 bobcat genotypes. Raw data for 102 bobcat genotypes using .gen extension for genepop.

10.7717/peerj.11498/supp-10 Supplemental Information 10 Geographic data for 102 bobcat samples. Raw data of XY coordinates for 102 bobcat samples.

10.7717/peerj.11498/supp-11 Supplemental Information 11 Environmental predictor variables for home range RDA. Proportions of each environmental variable within sample location buffer polygons at the home range spatial scale. For the linear variables (Stream Density, Railroad Density, and Road Density), we divided total variable lengths by the total hectares of the buffer polygons and converted to km/km2. For stream density, we included streams above an order of magnitude of 5 given the likelihood that anything below this cutoff would not be a significant barrier to bobcat movement. Maximum and minimum temperature reflected the weighted average of maximum minimum temperatures within each polygon. We calculated annual precipitation to a weighted average by intersecting this layer with buffer polygons and adding the products of the number of hectares at each rain average value (in) and divided by the total hectares of each buffer polygon.

10.7717/peerj.11498/supp-12 Supplemental Information 12 Environmental predictor variables for dispersal scale RDA. Proportions of each environmental variable within sample location buffer polygons at the dispersal range spatial scale. For the linear variables (Stream Density, Railroad Density, and Road Density), we divided total variable lengths by the total hectares of the buffer polygons and converted to km/km2. For stream density, we included streams above an order of magnitude of 5 given the likelihood that anything below this cutoff would not be a significant barrier to bobcat movement. Maximum and minimum temperature reflected the weighted average of maximum minimum temperatures within each polygon. We calculated annual precipitation to a weighted average by intersecting this layer with buffer polygons and adding the products of the number of hectares at each rain average value (in) and divided by the total hectares of each buffer polygon.

## Additional Information and Declarations

### Data Availability

The following information was supplied regarding data availability: R code for all statistical analyses is available in a Supplemental File.
