# Supplementary figures and images for: Multiscale patterns of isolation by ecology and fine-scale population structure in Texas bobcats
Source: PeerJ. 2021 Jun 3;9:e11498. doi: 10.7717/peerj.11498 (PMC8180196; doi:10.7717/peerj.11498)

**A****Spatial and variance components of the eigenvalues**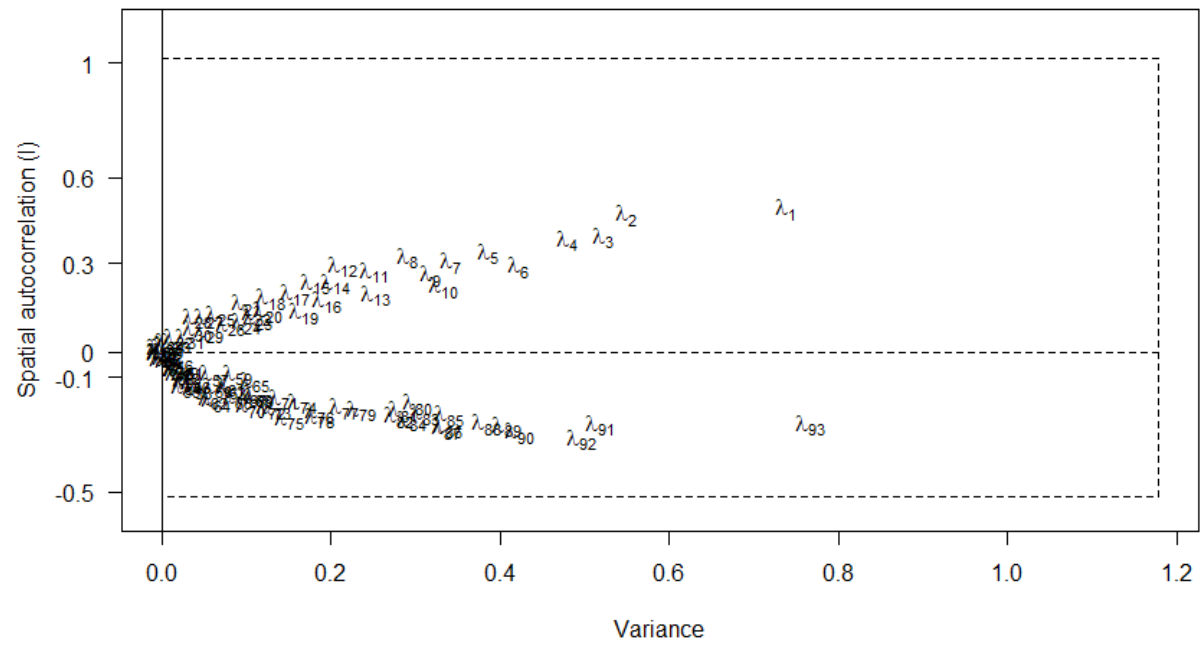**B**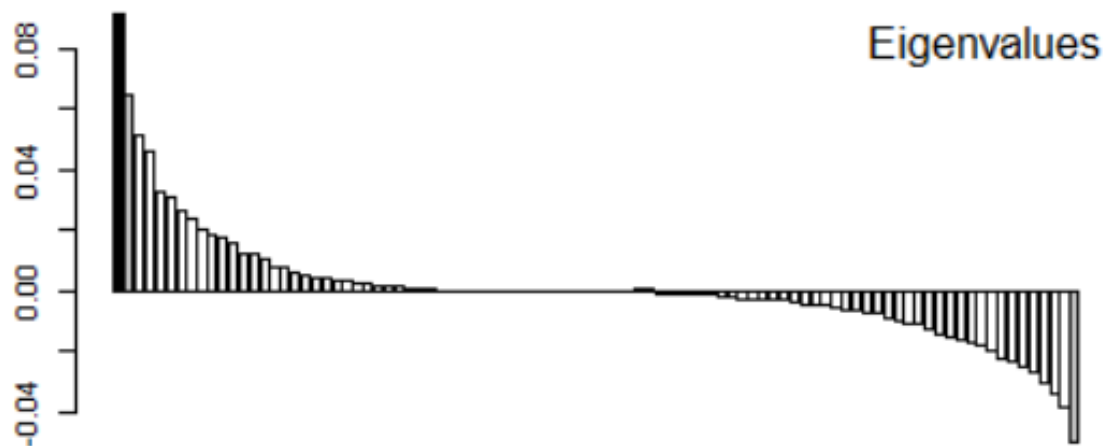

Supplement: Supplemental Information 1 — (A) Eigenvalues of sPCA (denoted λi with i = 1, …, r, where λ1 is the highest positive eigenvalue, and λ93 is the highest negative eigenvalue) according their variance and Moran’s I A B components. (B) Positive eigenvalues (on the left) correspond to global structures, while negative eigenvalues (on the right) indicate local patterns. [file peerj-09-11498-s001.pdf]

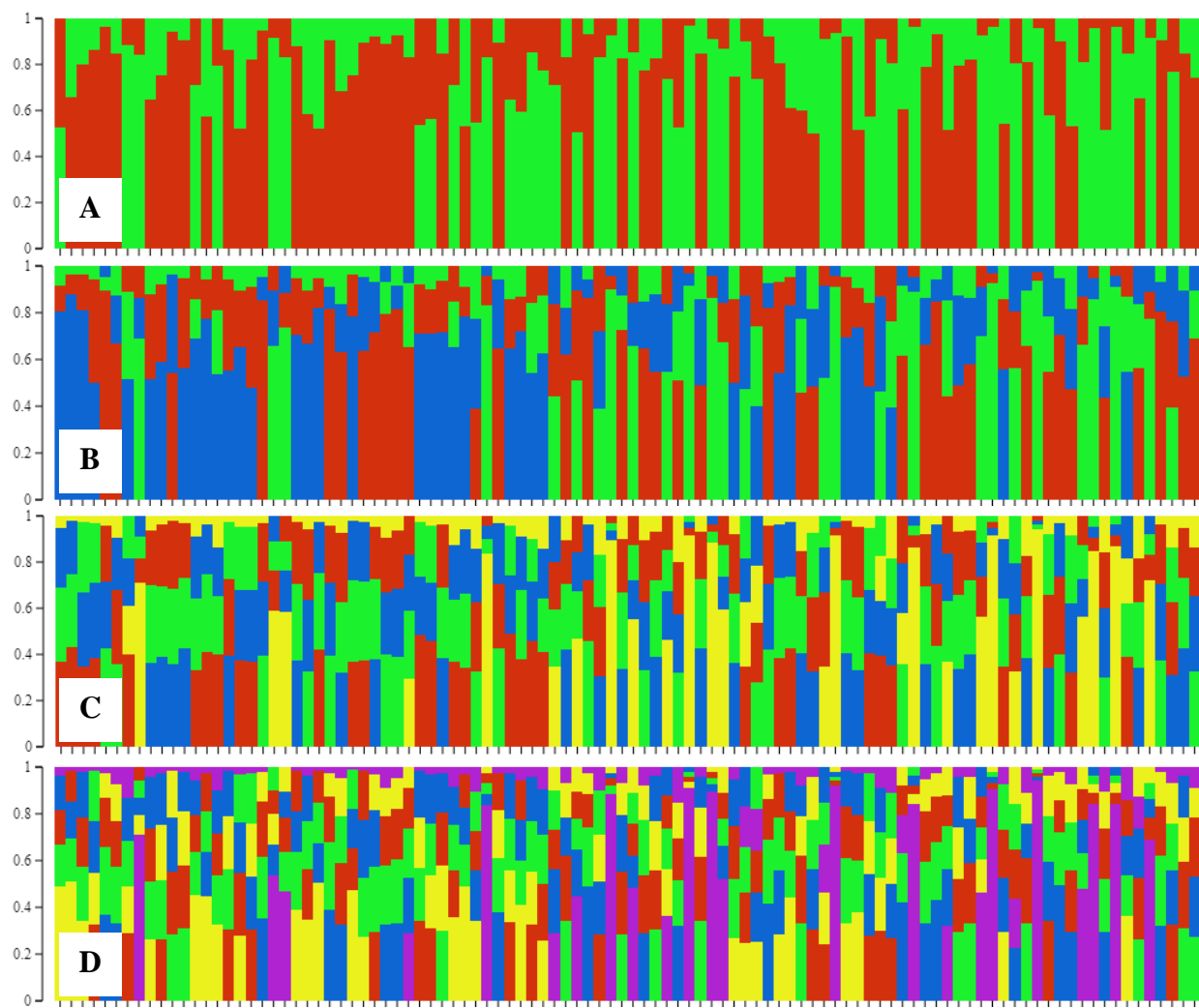

Supplement: Supplemental Information 2 — Structure of bobcat clusters (K) in western Texas revealed by Bayesian analysis implemented in STRUCTURE. Each individual is represented by a vertical bar broken into different colored genetic clusters and arranged by latitude, with length proportional to the assignment probability to each cluster. Analysis of 102 individuals, with possible numbers of clusters ranging from 2–10, indicated that the most likely number of clusters was 3. Bar plots created in STRUCTURE PLOT (Ramasamay et al. 2014). [file peerj-09-11498-s002.pdf]

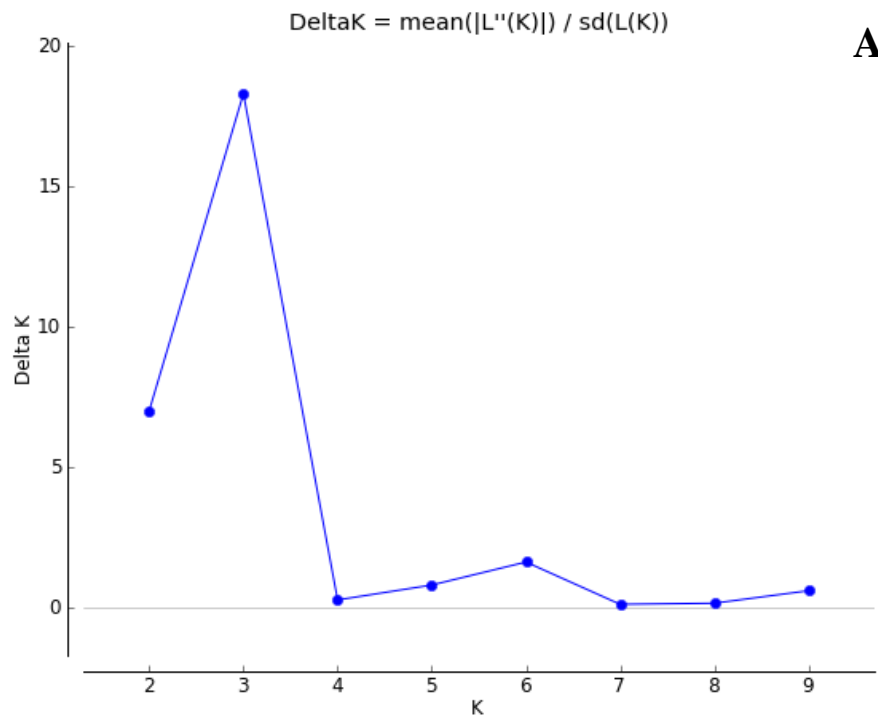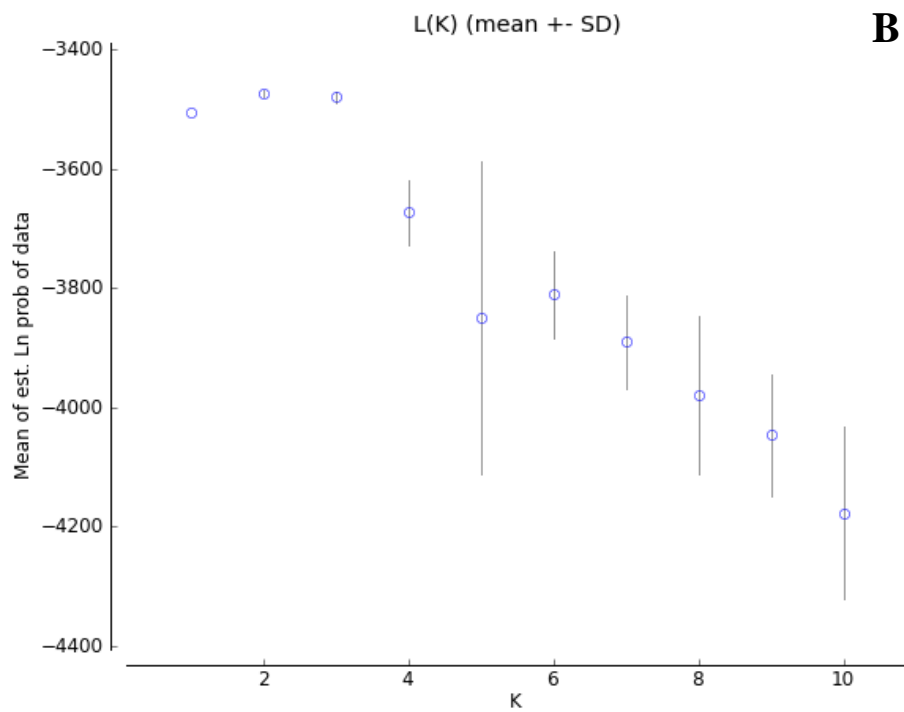

Supplement: Supplemental Information 3 — Statistical output from Structure Harvester suggesting [A] optimal K with ΔK and [B] LnP(D). [file peerj-09-11498-s003.pdf]

**Value of BIC  
versus number of clusters**

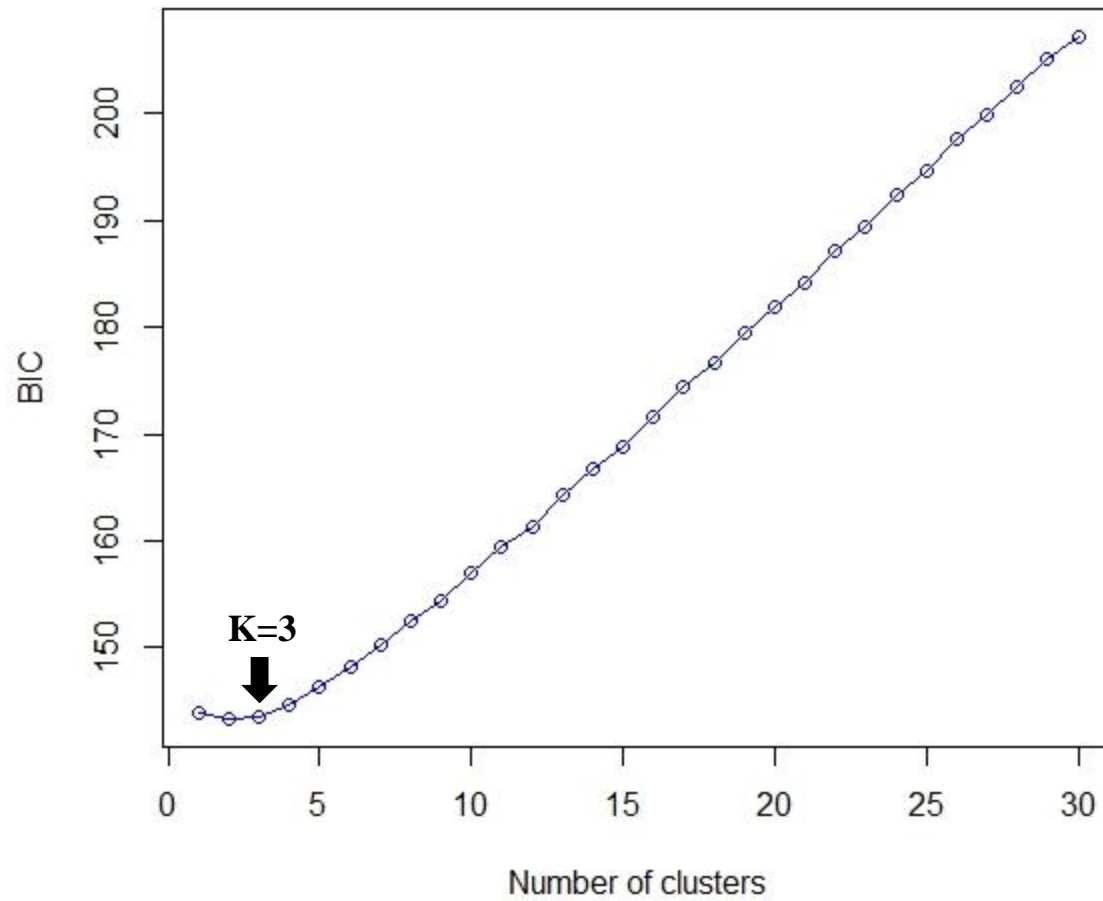

Supplement: Supplemental Information 4 — Bayesian information criterion (BIC) plot for DAPC between 1 and 10 showing an elbow with an arrow at K = 3. [file peerj-09-11498-s004.pdf]

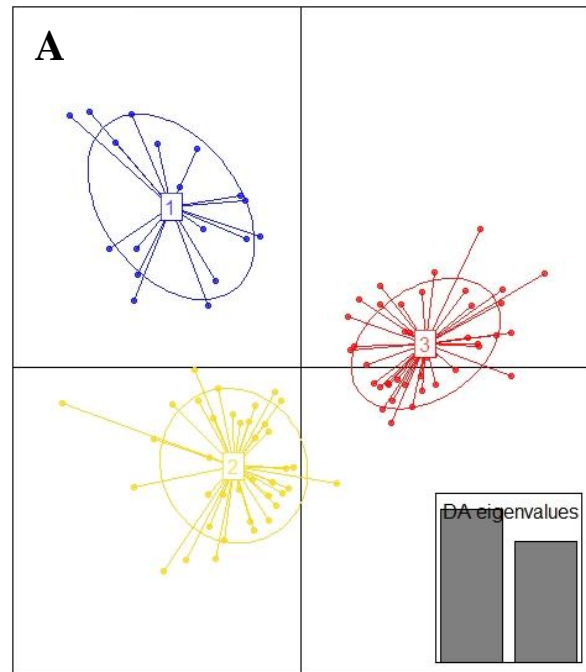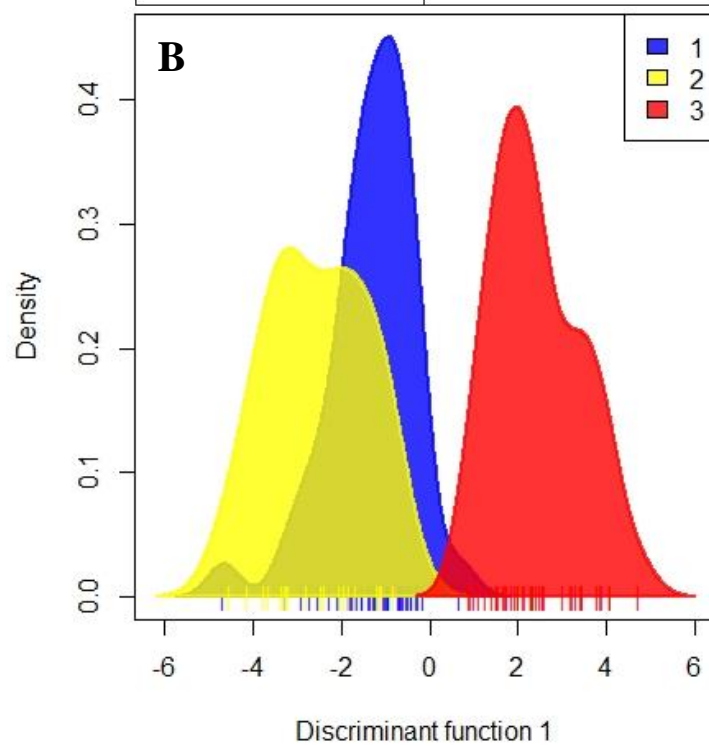

Supplement: Supplemental Information 5 — [A] Discriminant Analysis of Principal Components (DAPC) scatterplot drawn using nine microsatellites across 102 individual bobcats in the R package adegenet. Dots represent individuals, with colors denoting cluster assignment. [B] A plot of the individual densities against the first discriminant function retained show that the greatest proportion of variation lies with it. Colors correspond to cluster assignments, with significant overlap between clusters 1 and 3, suggesting two groups instead of 3. [file peerj-09-11498-s005.pdf]
