# Supplementary material for: Multiscale patterns of isolation by ecology and fine-scale population structure in Texas bobcats
Source: PeerJ. 2021 Jun 3;9:e11498. doi: 10.7717/peerj.11498 (PMC8180196; doi:10.7717/peerj.11498)
Supplement: Supplemental Information 6 — We intersected GIS layers for each variable with a buffer polygon at both spatial scales and calculated the proportion of each variable within each buffer polygon. For the linear variables (Stream Density, Railroad Density, and Road Density), we divided total variable lengths by the total hectares of the buffer polygons and converted to km/km2. For stream density, we included streams above an order of magnitude of 5 given the likelihood that anything below this cutoff would not be a significant barrier to bobcat movement. Maximum and minimum temperature reflected the weighted average of maximum minimum temperatures within each polygon. We calculated annual precipitation to a weighted average by intersecting this layer with buffer polygons and adding the products of the number of hectares at each rain average value (in) and divided by the total hectares of each buffer polygon. To determine which variables to use in the partial redundancy analysis (RDA), variables were selected from among nine environmental classes following Pearson’s correlation, and selected if r > 0.7. [file peerj-09-11498-s006.docx]

Table 1. Land use and environmental predictor variables retained for RDA at two spatial scales.

We intersected GIS layers for each variable with a buffer polygon at both spatial scales and calculated the proportion of each variable within each buffer polygon. For the linear variables (Stream Density, Railroad Density, and Road Density), we divided total variable lengths by the total hectares of the buffer polygons and converted to km/km2. For stream density, we included streams above an order of magnitude of 5 given the likelihood that anything below this cutoff would not be a significant barrier to bobcat movement. Maximum and minimum temperature reflected the weighted average of maximum minimum temperatures within each polygon. We calculated annual precipitation to a weighted average by intersecting this layer with buffer polygons and adding the products of the number of hectares at each rain average value (in) and divided by the total hectares of each buffer polygon. To determine which variables to use in the partial redundancy analysis (RDA), variables were selected from among nine environmental classes following Pearson’s correlation, and selected if *r* > 0.7.

| **Home Range Scale** | **Dispersal Range Scale** |
| --- | --- |
| Latitude | Latitude |
| Longitude | Longitude |
| Urban | Urban |
| Agriculture | Agriculture |
| Herbaceous Rangeland | Herbaceous Rangeland |
| Mixed Rangeland | Mixed Rangeland |
| Stream Density | Stream Density |
| **-** | Mesquite Juniper Shrub |
